# A Statistical Study on the Influence of Drilling Process in Delamination Observed in Composite Plates

**DOI:** 10.3390/ma18071595

**Published:** 2025-04-01

**Authors:** Hugo R. C. Cerqueira, João E. Matos, José L. Esteves, Susana C. F. Fernandes, Luis M. P. Durão

**Affiliations:** 1 ISEP, Polytechnic of Porto, rua Dr. António Bernardino de Almeida, 4249-015 Porto, Portugal; 1220546@isep.ipp.pt (H.R.C.C.); jem@isep.ipp.pt (J.E.M.); scf@isep.ipp.pt (S.C.F.F.); 2 INEGI Instituto de Ciência e Inovação em Engenharia Mecânica e Engenharia Industrial, 4200-465 Porto, Portugal; jesteves@fe.up.pt; 3 FEUP Faculdade de Engenharia da Universidade do Porto, 4200-465 Porto, Portugal

**Keywords:** composite materials, drilling damage, damage assessment, non-destructive testing, Taguchi method

## Abstract

Composite materials are increasingly being implemented in various solutions, ranging from conventional applications, like furniture, to more advanced ones, such as aerospace, based on their excellent properties, such as high mechanical strength and low weight. There are applications in which these materials are coupled to other parts. To achieve this connection, drilling processes are commonly used. Drilling causes irreversible damage to the material, which influences the mechanical strength of the plates. This study was conducted on 48 carbon/epoxy plates, each with two drilled holes, based on DOE (design of experiments) and the Taguchi method to design the experimental plan and to validate the results. Three control factors were considered for drilling: drill bit type, cutting speed, and feed rate, as it is expected that a low feed rate and a high cutting speed is the drilling configuration that inflicts the least damage. Subsequently, these specimens were subjected to enhanced radiography and an image analysis processing tool based on MatLab^®^ to assess the data collected and compute damage results. At the end, in analyzing the results of the Taguchi method, it is possible to validate the assumptions on the influence of the drilling process in delamination extension.

## 1. Introduction

In a progressively developed world, materials with enhanced properties are increasingly sought-after. These materials, like polymeric matrix composites, are widely used in diverse applications and industries. Examples of these applications are easy to identify, like the automotive industry [1,2], shipbuilding industry [3], wind turbines blades [4], or even sports [5] or furniture [6,7].

One of the advantages of these type of material is their excellent properties, such as high mechanical strength combined with low weight, resulting in a high specific strength. As a result of the way that parts are produced, they also provide a near-net shape, characterized by good surface finishing and no need for large machining processes. However, there are situations in which, due to the need to assemble structural parts, machining, especially drilling, is necessary. Drilling is a complex process that can cause significant damage, delamination being the most relevant, by negatively affecting the mechanical properties of the machined plates. This outcome of the drilling process and its consequences has been extensively studied by a considerable number of authors [8,9,10,11,12,13] and some significant revisions concerning advances in drilling techniques and delamination evaluation can be found in [14,15,16]. Delamination, which occurs at the entrance and exit of the hole, is one of the major damages to be studied.

### 1.1. Composite Plate Drilling

In drilling, material removal occurs due to the contact and rotation of the drill bit on the workpiece. Normally, drills have two cutting edges and two flutes to allow the easy removal of chips from the machining zone. The force that is exerted in the direction perpendicular to the part is called the axial thrust force (F_a_), and it is dependent on the plate material, the drill bit geometry and the drilling parameters. This axial thrust force is considered as primarily responsible for the damage caused in the drilling process. In [8], the authors investigated the influence of cutting speed, feed rate and tool diameter on the uncut fiber and delamination damage on composite sandwich structures using DOE (design of experiments), showing the influence of feed rate and the possibility of achieving an optimum point for cutting speed and tool diameter. In [9,10], similar conclusions on the importance of feed rate on damage were confirmed. Rajkumar et al. [11] conducted an experimental investigation using RSM (response surface methodology) to determine the influence of these parameters on the machining of composites, revealing the importance of drill diameter on delamination and thrust force. The influence of different drill geometries was the focus in [12,13], demonstrating the influence of tool geometry.

**Figure 1:**
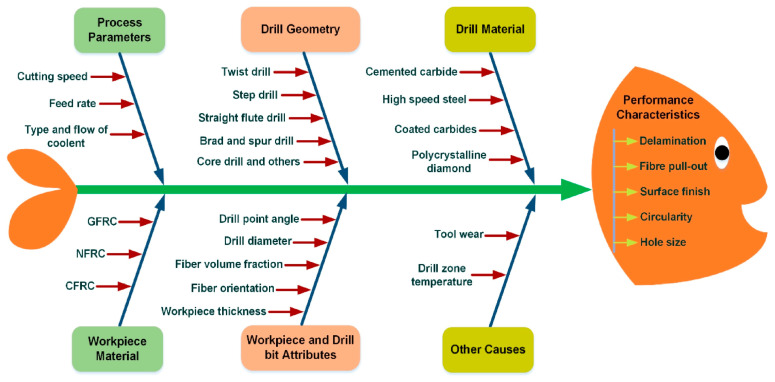
Cause and effect diagram showing the influence of various parameters on performance characteristics of drilled composites [15].

Figure 1 [15] outlines the main parameters that influence the drilling operation in composite materials. This figure summarizes the inputs that condition and alter the drilling for a composite material. In [14,15,16], it is possible to find reviews of the issues related to drilling of fiber-reinforced composites, including the minimization of delamination extension and the path to high-quality drilling, presenting the main findings of recent papers. The main parameters that influence conventional drilling are feed rate, cutting speed, and drill bit geometry. Of these parameters, that regarded as the most important is the feed rate as it is directly related to the axial thrust force, thus defining the damage caused by the drilling. Cutting speed is the second most important parameter among those mentioned above [10].

**Figure 2:**
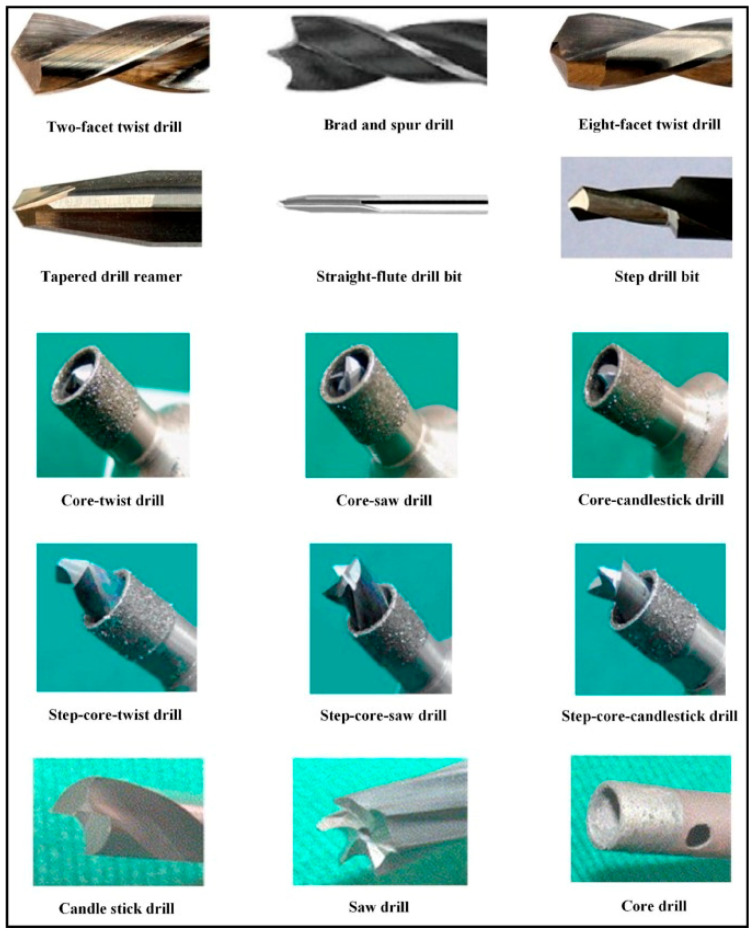
Various drill geometries employed in the drilling of composites [15].

Tool geometry also has some influence on the development of axial thrust force during drilling. Besides the standard twist drill, with variation of the point angle, defined as the angle formed by the two cutting edges of the drill bit, other drill geometries have been used when studying the drilling of composite plates, like Brad and Spur, step, straight-flute or core drill;, see Figure 2, showing some of the drills used to produce holes in composites [15]. The importance of drill point angle on the progress of axial thrust force and damage caused by drilling should be noticed. When using sharp-angled tools, lower cutting forces and less delamination damage is expected, whereas obtuse-angled tools register higher thrust forces, causing more delamination damage [17,18,19,20].

### 1.2. Non-Destructive Testing of Drilled Composite Plates

To achieve the necessary results in order to quantify and qualify the damage extension, it is necessary to analyze the region around the drilled holes with non-destructive techniques to determine the required geometrical parameters of the affected areas and, eventually, the mechanical properties of the materials after machining, ensuring the necessary strength [21,22,23,24]. The determination of mechanical properties of the drilled coupons is outside the scope of this study.

Non-destructive testing, or NDT, is particularly important in the evaluation and characterization of materials. Some of the NDT methods most commonly used in composite materials are radiography, ultrasound, eddy-current or tera hertz, among others. All these methods are suitable to detect delamination or other damage in the materials. When using radiography, it is normal to associate the use of liquid penetrant testing, by immersing the plate in a contrasting fluid. In enhanced radiography, a contrasting fluid is used to improve radiographic images. These liquids can help in the detection of small flaws in the interior of the material under analysis. As they are oriented perpendicularly to the radiation beam, delaminations are well detected by this method [25,26,27].

X-ray computed tomography (CT) has been increasingly used in composite materials as a technique for non-destructive testing. This technique allows for the reconstruction of the part, enabling highly accurate inspection of composite materials, as these materials are characterized by their heterogeneity and damage is sometimes difficult to carry out a proper assessment on. Therefore, 3D imaging can be used to analyze and evaluate the possibility of existing damages and thus ensure structural integrity of the parts. This technique is based on the computational reconstruction of a 3D image, obtained by the beams of radiation made from various angles of a part [28,29]. Examples of the use of this technique can be found in [30,31]

The ultrasound method is based on the incidence of high-frequency sound waves, between 20 kHz and 25 MHz, on the material to be analyzed. Due to the properties of composite materials, frequencies between 1 and 5 MHz are frequently applied. The most common defects detected in composite materials are fiber breakage, inclusions, matrix cracking and delamination [32].

Eddy-current can be adapted to characterize subsurface defects in composites, like delaminations, microcracks, porosity or fiber breakage, providing an effective and economical solution for the non-destructive inspection of CFRP [33,34].

As a final note for the use of Terahertz waves as NDT method, these waves use signal frequencies between 300 GHz and 3 THz, with wavelengths ranging from 1 mm to 100 μm. This method is characterized by using non-ionizing radiation to the detriment of ionizing radiation, such as X-radiation, assuring safety for the operator or avoiding consequences to biological samples. Terahertz demonstrates effectiveness in penetrating non-conductive materials, such as ceramics and plastics. The use of Terahertz as a non-destructive method has deserved the interest shown in recent research [35,36], as some advantages are easily recognized.

### 1.3. Image Processing Techniques for Delamination Assessment

**Figure 3:**
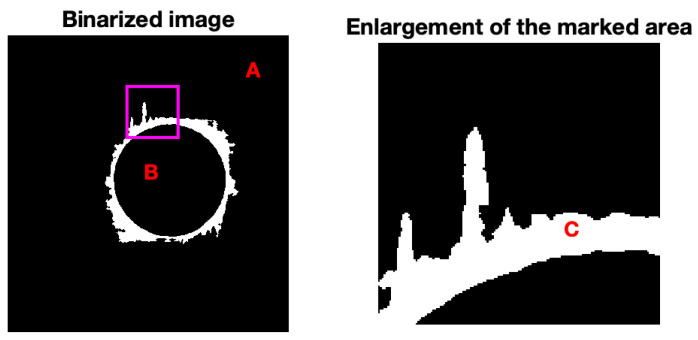
Example of a damaged area region: A (black outer region) is the undamaged coupon; B (black region) is the hole; C (white region) is the damaged area (full line).

A characteristic that is common to all these non-destructive methods is the recording of images to allow for posterior assessment of the geometrical features of the region around the drilled hole, as delamination criteria apply based on this. The processing and analysis of the images obtained during the process of radiography of the holes is a fundamental step towards the results of this dissertation. Image processing, based on MatLab^®^, version 23.2 or similar software, is a method frequently used to help on the quantification of the delaminated area around the hole, referred to by previous studies of the team involved in this work, such as Durão et al. [37] or Silva et al. [38]. The ultimate goal in any image processing method is to obtain images in which the pixels that correspond to the damaged area have one color, usually white, and the rest of the pixels in the same image another color, usually black (see Figure 3). The boundaries between the drilled region and the damaged area or between the damaged area and sound plate have various shades, and the same is true for the damaged area, the drilled area or the plate surface. It is therefore necessary to define a strategy that permits a clear definition of the contours and areas. A technique often used for this purpose is the use of threshold. Threshold is an algorithm that calculates an edge value, which divides the grayscale values, turning the image into a binary image. In other words, the algorithm assigns a value to each color in the image, between 0 and 255, calculates an average value of the grayscale of an image and assigns a black pixel for values below the defined threshold and a white pixel for values above it, resulting in a black-to-white image, with sharper contours and areas. Several software applications that allow image processing already have the threshold tool incorporated, as is the case with Matlab^®^, used in the study here presented.

Alternative approaches to the assessment of damaged area are the use of ANN (Artificial Neural Networks) or even the use of AI tools, considering the increased computational capacity of modern computers. Both can be used for any process where the starting point is an image obtained by some NDT method, as in those mentioned in 1.2. Concerning ANN, this research team has presented a novel solution based on an artificial neural network in the analysis of radiographic images [39]. In [40], recurrent neural networks (RNNs) were developed and implemented to estimate tool wear during composites drilling.

The study of delamination requires quantifying it in some way. Only in this way is it possible to assess how the various factors already mentioned can affect delamination, to establish comparisons between them and to seek solutions to mitigate this phenomenon. The evaluation of delamination begins with obtaining some kind of image of the drilled hole and its peripherical region [38]. Afterwards, it is fundamental to define some mathematical criteria for this assessment. One of the early criteria presented was the Delamination Factor (F_d_) [41], Equation (1), which is obtained by the ratio between the maximum diameter of the damaged area (D_max_) and the nominal diameter of the hole (D_0_).

(1) $$F_{d}=\frac{D_{max}}{D_{0}}$$

**Figure 4:**
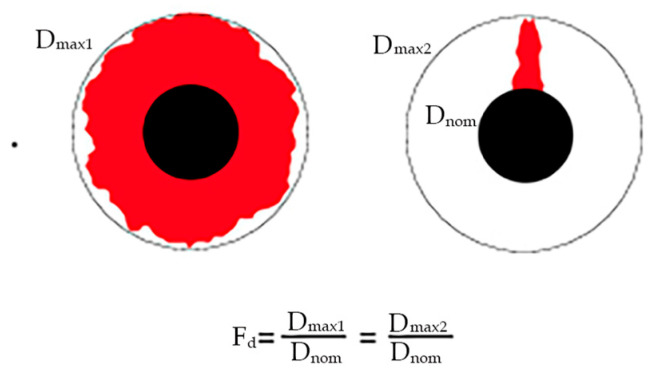
Example of equal delamination factors for different damaged areas (damage area is marked in red) [42].

However, this factor has the limitation of being one-dimensional, not considering the effect of the damaged area, so two holes with different areas of damage can have the same value of F_d_ (see Figure 4) [42]. Additionally, in cases where the damaged area is more irregular and not circular, it is advisable to use the damaged area to quantify delamination instead of the maximum diameter.

Several solutions have been proposed to overcome this problem (see Davim et al. [43]), which suggested the Adjusted Delamination Factor, Equation (2):

(2) $$F_{da}=\alpha \frac{D_{max}}{D_{0}}+\beta \frac{A_{max}}{A_{0}}$$

where A_max_ is the damaged area and A_0_ the nominal hole area.

**Figure 5:**
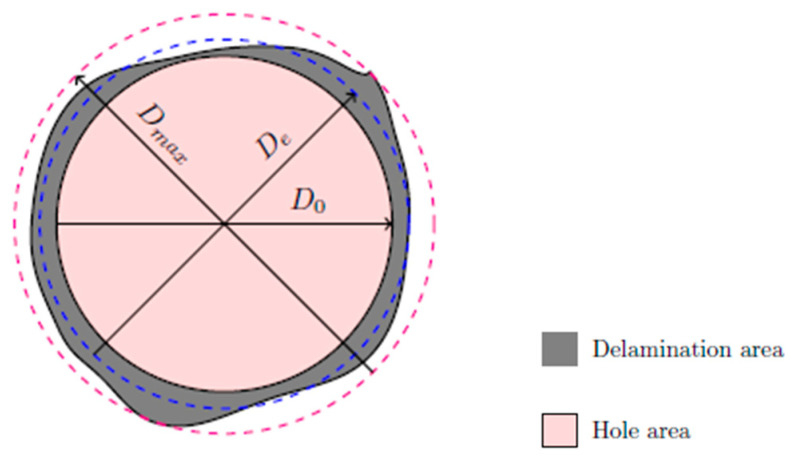
Scheme for Equivalent Delamination Factor.

Recently, Tsao et al. [44] developed the Equivalent Delamination Factor, (F_ed_), which relates an equivalent diameter (D_e_), see Figure 5, to the nominal diameter of the hole (D_0_), Equations (3) and (4). This criterion was adopted for the study here presented.

(3) $$F_{ed}=\frac{D_{e}}{D_{0}}$$

(4) $$D_{e}={\left(\frac{{4(A}_{d}+A_{0})}{\pi }\right)}^{0.5}$$

A tridimensional delamination criterion (F_v_) was recommended by Xu et al. [45] and it would be interesting to consider this factor in this work, as it includes the accumulated volume of the various delaminated plies along the plate, Equation (5), where p is the number of layers and k is the number that specifies the delaminated ply. However, for the current state of the radiographic setup, this option was not possible to incorporate, remaining as a challenge to meet in future work.

(5) $$F_{v}=\frac{1}{p}\sum_{k=1}^{p}\frac{A_{d}^{k}}{A_{nom}}$$

Therefore, understanding and predicting how the drilling process can affect the damage extension and, consequently, the mechanical strength of composite plates is the main purpose of this study. The use of the Taguchi method is helpful in designing the experimental sequence and helping with a sound analysis of the results obtained regarding damage evaluation and correlations with the experimental factors defined for this study.

## 2. Materials and Methods

**Figure 6:**
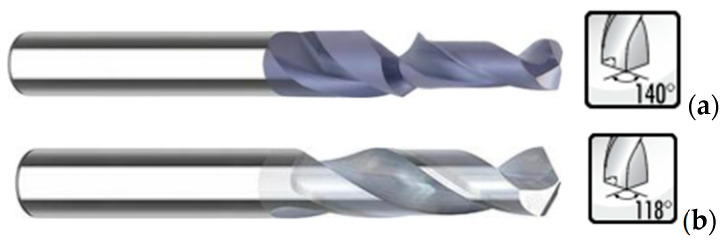
Tools used in experimental work (Source: INOVA Tolls catalogue): (**a**) step drill; (**b**) twist drill.

To evaluate the effect of drilling on composite materials and considering the information collected in the bibliographic review, the main parameters of drilling to include in this study were decided: feed rate, cutting speed and drill bit geometry. Two possible levels have been assigned to each of these characteristics. For feed rate, the values of 0.05 and 0.2 mm/rev were established. The choice of these values was based on bibliographic research and analysis of previously published papers in the same field [13,14,15,16,17,21,22,37,38,46]. These values are within a range of acceptable drilling values for the composite material and from which satisfactory results are predictable. For the cutting speed, the decision was to use spindle speed values of 500 and 2000 rpm, corresponding to 9.4 and 37.7 m/min. For the 6 mm drill geometry, a step drill from INOVA Tools (Kinding, Germany), Ref. 850.037.00, with a first diameter of 3.7 mm and a point angle of 140° was chosen (see Figure 6a). When using a step drill, it is reported that larger feed rates can be used, meaning shorter cycle times, without delamination damage [46]. The other option was the use of a twist drill in a pilot hole drilling sequence, using two twist drills of different diameters, with a ratio of 0.4 from the pilot to the final hole, following a previous published study [47]. The advantage of pilot hole drilling on delamination reduction by cancelling the chisel edge effect during final diameter machining was evidenced in [48,49]. For the twist drills, the point angle was 118°. These tools were also from INOVA Tools, Refs. 701.024.000 for the pilot hole and Ref. 701.060.000 for the final hole (see Figure 6b). The drills selected are capable of drilling composite materials and are within a range that has normally been evaluated in this type of study.

A summary of the experimental levels and their unfolding for experimental work is provided in Table 1.

With the help of Minitab^®^ software, version 22.1.0, a DOE (design of experiment) was created for the application of the Taguchi method, resulting in two different plans. One is simpler, with only four configurations of the control factors, which results in a sample array of four, which was abandoned. Another DOE was prepared, with all the configurations of the three factors, which resulted in an array of eight, as illustrated in Table 1, together with the identification adopted for the plates drilled in each option. Drill bit wear was considered as the noise factor; therefore, the planning was repeated to check and verify the results.

**Figure 7:**
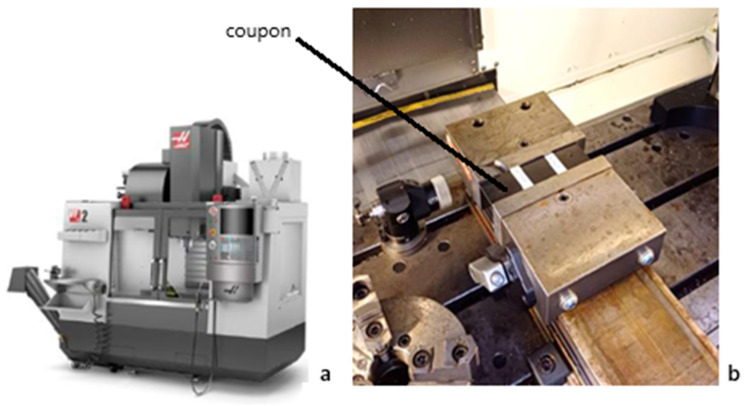
(**a**) HAAS VF-2 CNC machine (HAAS, Oxnard, CA, USA); (**b**) Image of a drilled coupon.

To run this experimental plan, four laminate plates were produced, each consisting of twelve layers of prepreg, symmetrically stacked and balanced. The laminate was of the cross-ply type, and the stacking sequence was [0°/90°]_3s_. The plates were produced using a HEXCEL prepreg from HEXCEL^®^, Stamford, CT, USA, composed of AS4 12K carbon fibers (HexTow^®^) and 8552 epoxy resin (HexPly^®^). The prepreg has a nominal fiber volume of 57%. Material characteristics of the prepreg material can be seen in Table 2. The plates produced were approximately 300 mm wide and long and 2.2 mm thick. Before drilling, plates were cut in coupons of the appropriate dimension for the testing sequence planned, considering the coupons needed for the complete DOE. A total of 12 holes were drilled at each level, including the necessary repetitions for statistical analysis soundness, and tool wear evaluation, only at two levels, new and worn. Machining was performed in a HAAS VF-2 machining center (Figure 7a,b).

Then, a non-destructive alternative was determined, in this particular case enhanced radiography, since this had already been used in previous works, reducing uncertainty in results [37,38,50]. For enhanced radiography, coupons were immersed in diiodomethane, a contrasting liquid, for 15 min and then radiographed with the help of a digital imaging system consisting of a 60 kV, 300 kHz Kodak 2100 X-ray system (Kodak, Rochester, NY, USA) associated with a Kodak RVG 5100 digital acquisition system. The exposition time was set to 0.25 s [50]. Following the line of this study, the images obtained by enhanced radiography were analyzed and treated using a program developed in MatLab^®^. This software was used to convert radiographic images into binary images composed of black and white pixels. From this point, the program calculated the values of the geometric parameters that are understood as useful, knowing that each pixel corresponds to a square with a side of 0.0185496 mm.

As already stated, the delamination criteria used in this study was the Equivalent Delamination Factor, (F_ed_) [45] where the delamination assessment is computed from the measurement of the delaminated area resulting from the use of a computational routine developed on MatLab^®^ for radiographic image treatment, following Equations (3) and (4) (see Section 1) and enabling the values of the delaminated area around the hole. The minimum value of this factor is 1 (one), equal to a situation where no delamination is observed, or greater than 1 (one) if some delamination is observed. Inherently, the greater the value of this factor, the greater the delaminated area.

Finally, the application of the Taguchi method was completed to validate the results and identify the best drilling configuration. With this method, it is also possible to evaluate the interactions that the different factors have with each other and the significance that each of them represents for the result. The drill bit wear was considered a noise factor. Therefore, the values of this factor were inserted for new and worn drills.

To analyze the results obtained by this method, it is necessary to identify the best level for each control factor. Through the S/N ratio (signal/noise), the configurations of the control factor that minimize the variability caused by the noise factor are identified. In the linear analysis of the model, several coefficients are calculated, the most important being the p-factor. This is calculated for each control factor and for the interactions between each of the factors considered. The p-factor determines the statistical significance of each control factor in the response. It is usually assigned a significance level α of 0.05, which indicates a 5% risk of concluding that there is a significance when in fact this does not exist. If the *p*-value is less than or equal to the significance level, it is possible to conclude that the factor in question is statistically significant for the response. If the *p*-value is greater than or equal to the level of significance, it is not possible to conclude that there is significance among the experimental factors involved [51,52].

## 3. Results and Discussion

### 3.1. Preliminary Testing

As a starting point, for confirmation of plate properties, a tensile test according to ISO 527-1:2019 [53] was performed. As the relevant properties considered were the elastic modulus and the tensile strength of the material, a test speed of 1 mm/min was adopted until a load of 8 kN was reached and then switched to 2 mm/min. to provide data for both properties. A summary of the results is presented in Table 3, concerning the data from 8 tests.

During the drilling phase, no data were monitored regarding axial thrust force, as there are previous studies confirming the effect of tool geometry, cutting speed or feed rate on this outcome.

### 3.2. Delamination Measurement

The enhanced radiographic analysis process, described in the previous section, permitted quantification of the damage caused by the drilling operation. For that purpose, the plates were immersed for 15 min in diiodomethane, a contrasting fluid, before image capturing. Through the image processing sequence, it was possible to obtain the following results (see Table 4) that represent the average of 6 coupons under each drilling condition.

By analyzing the average values calculated, the holes with the pilot hole strategy return lower values for the damaged area, meaning that less damage was caused by drilling.

Taking all the values obtained from the image treatment of the radiographs, it was possible to calculate the equivalent delamination factor for every situation, using the criteria as defined in Section 2 (see [44] and Equations (4) and (5)), where the relevant values in Table 4 are the damaged area and the nominal dimensions of the drilled hole. Resulting F_ed_ average values are also presented in Table 4.

By analyzing these results, it is possible to conclude that a higher cutting speed and a lower feed rate cause less damage, as the higher cutting speed is the common factor in the two best values shown in Table 4.

From the combination of these factors for both drilling strategies, it results that the configuration that has a higher equivalent delamination factor is that with a high feed rate and a low cutting speed, using a step drill geometry. On the other hand, the drilling operation that results in a lower equivalent delamination factor is that which results from using a pilot hole strategy with a low feed rate and a high cutting speed, considering the range of this experimental study. Lower feed rates reduce the thrust force during drilling, keeping these values below the threshold for delamination onset, as demonstrated in [54]. Higher spindle speeds avoid long contact between the cutting edges of the drill and the hole walls, reducing temperature during drilling, which prevents matrix softening and, consequently, delamination or other related damages increase in temperature during dry drilling, reduce the elastic modulus of the CFRP and cause thermal expansion of the drill [13,55].

Based on previous knowledge of the delamination effects on mechanical features of plates [13,22,38,48,56], an increase in the F_ed_ value represents a plausible decrease in the bearing strength resulting from the Bearing test (ASTM D5961 [57]), or another mechanical test with the same objective. This trend is normally expected, meaning that a higher value of F_ed_ represents greater damage around the hole that, in turn, causes a greater loss of the mechanical strength of the plate. The completion of destructive confirmation tests is out of the scope of this study.

### 3.3. Taguchi Method Analysis

Finally, the application of the Taguchi method was completed to validate the results of this experimental design and identify the best drilling configuration among the options considered in this study. With this method, it is also possible to evaluate the interactions that the different factors have with each other and the significance that each of them represents for the result. The data for the equivalent delamination factor (F_ed_) was then analyzed. For that purpose, drill wear was considered as a noise factor. Therefore, the values of this factor were inserted for new and worn drills. For worn drills, new drills equal to those used in the first experimental step were used to produce 48 consecutive holes in a sacrificial plate identical to those of the experimental sequence.

For the F_ed_ analysis, the ratio chosen for the S/N ratio was “lower is better”, because the objective of the experiment was to minimize the response, which in this case is the F_ed_ value. Although the S/N ratio was considered as “lower is better”, the choice of optimal levels should be made in such a way that the S/N ratio value is maximum. This is because minimizing the loss-to-function is associated with maximizing the S/N ratio; therefore, the higher the value of this ratio, the better. Table 5 and Table 6 represent the linear analysis for the equivalent delamination factor average value and for the correspondent S/N ratios.

By analyzing the values in the tables above, it is possible to conclude that for the F_ed_ average, the drill type and the cutting speed are significant as they have a p-factor less than 0.05. However, the lowest value is that of cutting speed, so this control factor is the most significant, followed by the drill type. The feed rate factor has a value of 0.051, which makes it not significant, nonetheless this value is very close to the limit. For the interactions between the control factors, all of them have a p-factor greater than 0.05, which means that they do not have statistical significance for the average calculated for F_ed_. Regarding the S/N ratios, the p-factors of the control factors are all significant for the result. On the other hand, the interactions between them are not statistically significant.

**Figure 8:**
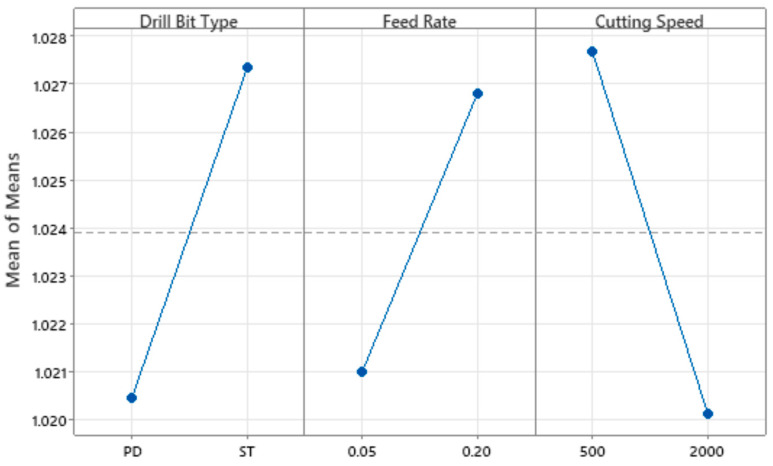
Average values—main effects of experimental factors.

**Figure 9:**
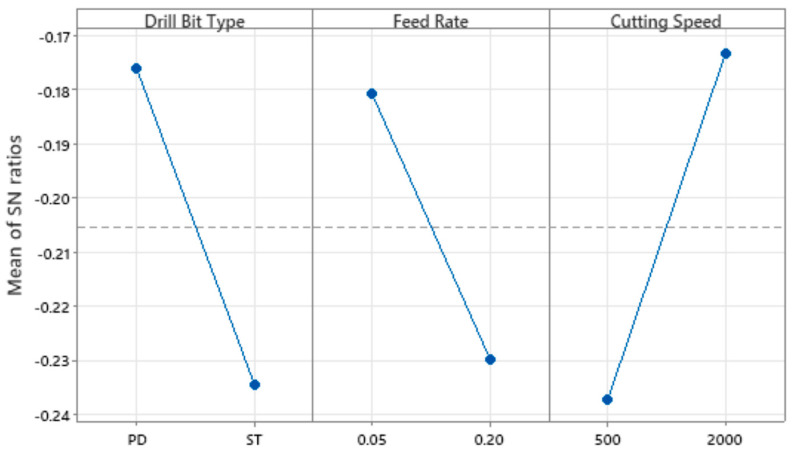
Signal-noise ratio of experimental factors.

For a better interpretation of these results, it is possible to elaborate several graphs from the analysis software, as illustrated below, see Figure 8 and Figure 9.

As previously stated, the objective is to maximize the value of the S/N ratio. The drilling setup that allows this premise employs a pilot hole strategy using a standard twist drill with a 140° point angle, a feed ratio of 0.05 mm/rev and a spindle speed of 2000 rpm, equal to a cutting speed of approximately 38 m/min when a 6 mm diameter drill is used. Note that different drill diameters can turn into diverse conclusions.

The concern of drilling with a new or with a worn drill bit was also compared for F_ed_. Therefore, it is possible to say that the values of the equivalent delamination factor are lower in drilling with the new drill than with the worn one. The worn drill is also associated with higher standard deviations of damage extension, characteristic of a more irregular drilling.

Finally, and to validate the results, the Taguchi method was used, and it was possible to identify which control factors were most significant and evaluate the interactions between them. For the F_ed_ data, we can conclude that all factors are significant, with the cutting speed as the most significant factor, and that the ideal drilling configuration is the one performed with a pilot hole strategy, with a feed rate of 0.05 mm/rev and a spindle speed of 2000 rpm.

## 4. Conclusions

This work aimed to study the effects of drilling on the damage extension of a composite plate by using enhanced radiography, an image processing tool based on MatLab^®^, and the Taguchi method. With this purpose, several drilling tests were carried out to characterize the damage around the drilled holes under diverse conditions of drill geometry, feed rate and cutting speed.

The analysis by enhanced radiography permitted to quantify the damage extension caused by drilling, calculating the equivalent delamination factor (F_ed_).

The application of Taguchi method was crucial to validate the results and identify the best drilling configurations. It was concluded that the combination of pilot hole drilling, feed rate of 0.05 mm/rev and a spindle speed of 2000 rpm provided the best results in terms of lower drilling-induced delamination extension. Statistical analysis showed that spindle speed and the drill geometry are significant factors, while feed rate has a lower significance.

This study demonstrated that the drilling parameters can influence damage extension in composite materials. Through a detailed experimental approach and the application of statistical methods, such as the Taguchi method, it was possible to identify the drilling configurations that could improve the quality of the holes.

Future work should include the use of computed tomography (CT-scan), making it possible to apply a three-dimensional delamination factor and, consequently, obtain more accurate results. Another possible improvement would be the use of AI (Artificial Intelligence) regarding the definition of the threshold value in image processing.

Other works could include similar studies with different composite materials, varying the number of layers and the stacking sequence or the fiber direction, thus contributing to an improved understanding of the mechanical behavior of composite materials.

## Figures and Tables

**Table 1:** Experimental plan—parameters levels.

Coupon ID	Drill Geometry	Feed Rate mm/rev	Spindle Speed rpm
1–PD0505	Pilot hole drilling	0.05	500
2-PD0520	Pilot hole drilling	0.05	2000
3-PD2005	Pilot hole drilling	0.20	500
4-PD2020	Pilot hole drilling	0.20	2000
5-ST0505	Step drill	0.05	500
6-ST0520	Step drill	0.05	2000
7-ST2005	Step drill	0.20	500
8-ST2020	Step drill	0.20	2000

**Table 2:** Prepreg mechanical properties—HexPly^®^ 8552 UD Carbon Prepregs, in HEXCEL, USA.

Test	Units	Value
0° Tensile Strength	MPa	2207
90° Tensile Strength	MPa	81
0° Tensile Modulus	GPa	141
90° Tensile Modulus	GPa	10
0° Compression Strength	MPa	1531
0° Compression Modulus	GPa	128
0° ILSS	MPa	128
In-plane Shear Strength	MPa	114

**Table 3:** Average mechanical properties of the plates.

Test	Units	Average	Std Dev
Tensile Strength	MPa	971.44	82.09
Tensile Modulus	GPa	66.55	2.34

**Table 4:** Measured hole and delamination values.

Coupon ID	Hole Area	Damage Area	F_ed_
1-PD0505	28.227	1.312	1.023
2-PD0520	28.456	0.759	1.013
3-PD2005	28.566	1.552	1.027
4-PD2020	28.547	1.097	1.019
5-ST0505	28.248	1.694	1.029
6-ST0520	28.365	1.154	1.020
7-ST2005	28.492	1.832	1.032
8-ST2020	28.571	1.121	1.028
Average PD	28.449	1.180	1.022
Average ST	28.419	1.450	1.028

**Table 5:** Analysis of variance for experimental results.

Source	DF	SQ Seq	SQ(aj)	QM(aj)	F	*p*
Drill type	1	0.000094	0.000094	0.000094	214.36	0.043
Feed rate	1	0.000067	0.000067	0.000067	152.60	0.051
Cut speed	1	0.000113	0.000113	0.000113	257.40	0.040
Drill*Feed	1	0.000000	0.000000	0.000000	0.98	0.504
Drill*speed	1	0.000004	0.000004	0.000004	9.09	0.204
Feed*speed	1	0.000005	0.000005	0.000005	10.86	0.188
Residual error	1	0.000000	0.000000	0.000000		
TOTAL	7	0.000284				

**Table 6:** S/N ratio variance analysis.

Source	DF	SQ Seq	SQ(aj)	QM(aj)	F	*p*
Drill type	1	0.006808	0.006808	0.006808	231.74	0.042
Feed rate	1	0.004790	0.004790	0.004790	163.06	0.050
Cut speed	1	0.008221	0.008221	0.008221	279.83	0.038
Drill*Feed	1	0.000031	0.000031	0.000031	1.04	0.494
Drill*speed	1	0.000302	0.000302	0.000302	10.28	0.192
Feed*speed	1	0.000364	0.000364	0.000364	12.38	0.176
Residual error	1	0.000029	0.000029	0.000029		
TOTAL	7	0.020544				

## Data Availability

The original contributions presented in this study are included in the article. Further inquiries can be directed to the corresponding author.
